# Embolization of Vessels With Irregular Lumen Using the Coil Packing Technique Between the Amplatzer Vascular Plug II Lobes

**DOI:** 10.7759/cureus.60469

**Published:** 2024-05-16

**Authors:** Masafumi Kaiume, Ryo Kurokawa, Toshiyuki Unno, Yoshifumi Nishino, Takuya Miyahara

**Affiliations:** 1 Department of Radiology, The University of Tokyo, Tokyo, JPN; 2 Department of Radiology, Showa General Hospital, Tokyo, JPN; 3 Department of Cardiothoracic Surgery, Showa General Hospital, Tokyo, JPN

**Keywords:** interventional radiology-guided embolization, aortic stenosis, pelvis cavity, coil packing, amplatzer plug device

## Abstract

Introduction: The Amplatzer Vascular Plug (AVP) series enables precise positioning and high migration resistance, allowing embolization in short segments; however, inadequate embolization or recanalization may occasionally occur. We hypothesized that leaks may occur when AVPs are implanted in vessels with irregular lumen due to insufficient adherence to the vessel. This hypothesis was tested by experiments with a vascular model. We employed a coil packing technique between the AVP lobes to embolize internal iliac arteries with an irregular lumen.

Methods: Saline was injected through the Y-shaped glass tubes of the stenotic and the smooth model (without stenotic lesion), and the amount of leakage was measured when the AVP was deployed. The feasibility and effectiveness of filling coils between the lobes of AVP II were evaluated. A total of 11 cases were retrospectively reviewed using this technique for internal iliac artery embolization prior to endovascular aortic repair.

Results: The amount of leakage was significantly higher in the presence of stenotic lesions. Insertion of a 2.2 F microcatheter from the side of the proximal lobe of AVP II and filling of coils was achieved in all 11 cases. Follow-up contrast-enhanced CT showed no recanalization, leakage, or other obvious complications.

Conclusion: Coil packing technique around Amplatzer Vascular Plugs could be an effective method and a reliable option for arterial embolization, especially in vessels with irregular lumens.

## Introduction

Vascular embolization is a treatment that involves the placement of embolic materials within blood vessels to obstruct blood flow, which also aids in promoting localized thrombosis, thus occluding the target vessel. This technique is utilized for the treatment of bleeding, aneurysms, arteriovenous fistulas, hypervascular tumors, blood flow modification procedures, and other applications. In the treatment of aneurysms, there are two types of procedures as follows: packing, which involves the placement of embolic material within the aneurysm, and isolation, which completely obstructs the blood flow entering the aneurysm. Isolation is commonly chosen for treating large aneurysms, such as those of the internal iliac artery.

The Amplatzer Vascular Plug (AVP; Plymouth, MN: Abbott Vascular) has been widely used as an embolization device for large-diameter vessels [1,2]. The AVP uses nitinol mesh to occlude blood vessels by facilitating blood clot formation, which can embolize short segments of vessels with a low risk of migration into distal branches [3]. However, embolization of high-flow vessels with AVP alone has a risk of residual flow or recanalization [4,5]. To overcome this limitation, the coil in plug (CIP) technique has been reported as a method for reliable embolization of short segments [6-9]. This technique, which achieves a reliable embolic effect over a short segment, is innovative and has become widely used in recent years. However, when using this technique for vessels with irregular lumens, especially with heavy irregular calcification, another problem can arise, i.e., AVP may not adhere well to the vessel wall. To overcome this disadvantage, we employed a coil packing technique (macaron technique) between the AVP lobes to embolize internal iliac arteries (IIAs) with irregular lumens in 11 patients after a preliminary experimental study.

## Materials and methods

This single-center retrospective study was approved by the Showa General Hospital Ethics Committee (approval number: REC-292) and conducted in accordance with the ethical standards laid down in the 1964 Declaration of Helsinki and its later amendments. Written informed consent was waived due to the retrospective design of the study.

**Figure 1:**
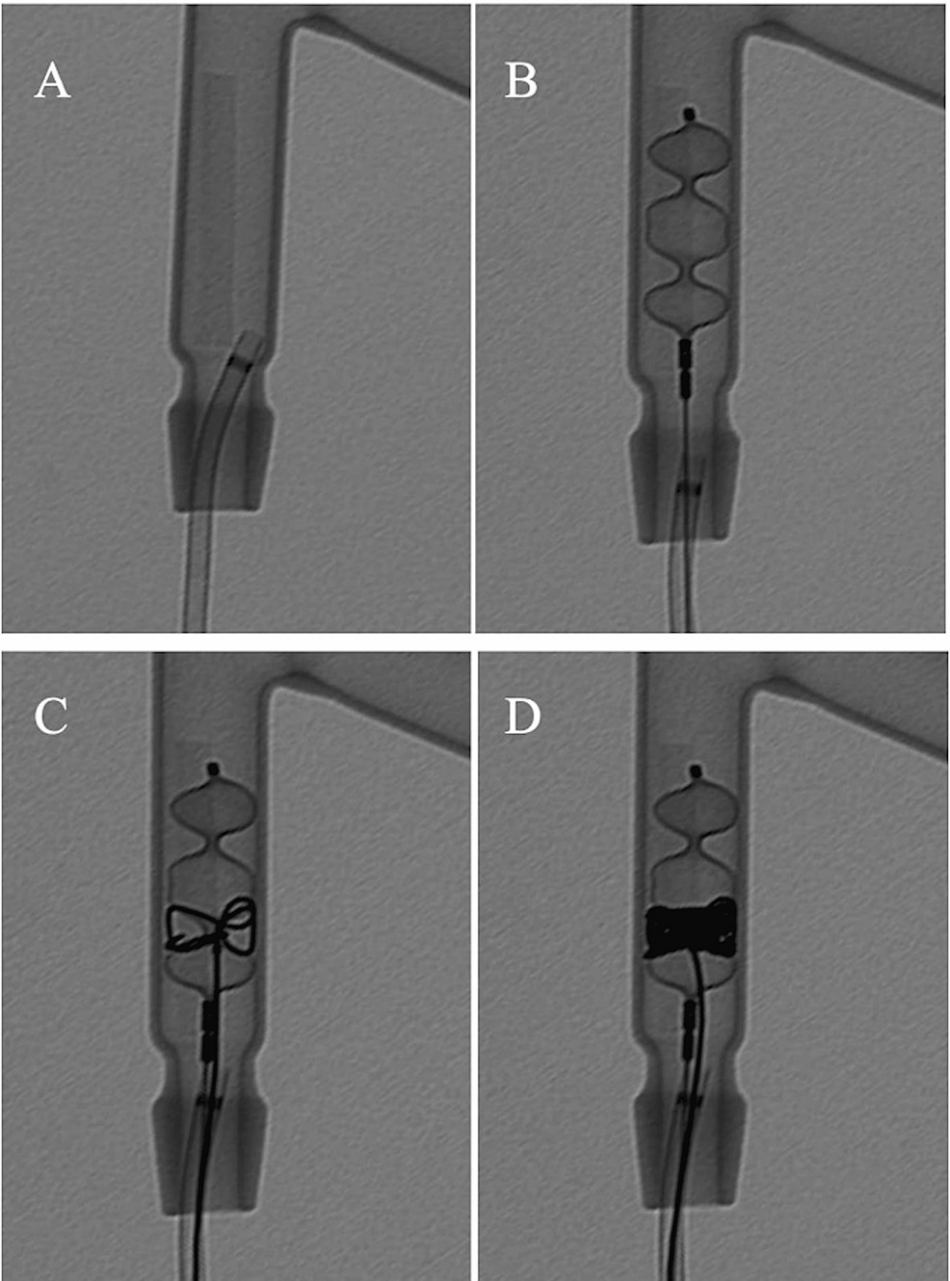
Experimental vessel model and AVP II. Experimental vessel model with stenotic lesion (A), AVP II deployed in the vessel model (B), and microcatheter inserted between the lobes of AVP II and filled with microcoils (C, D). AVP: Amplatzer Vascular Plug

A stenotic lesion model was created by gluing 2 mm diameter thick soft polyvinyl chloride to a Y-shaped glass tube with a lumen diameter of 6 mm (the stenotic model) (Figure 1A). A simulated method of coil packing technique between the AVP lobes was performed under direct fluoroscopic visualization of the vascular model. The AVP II 8 mm was inserted into the stenotic lesion from the A 5 F guiding sheath (Destination; Tokyo, Japan: Terumo Corporation) and deployed (Figure 1B). Before detaching, a 2.1 F microcatheter (Nadeshiko, Hiroshima, Japan: JMS Co., Ltd.) and a 0.016-inch microwire (ASAHI meister 16; Seto, Japan: Asahi Intecc Co Ltd) were inserted coaxially from the guiding sheath, and microcatheter was inserted between the proximal and central lobes from the side of the proximal lobe of the plug (Figure 1C). A microcoil (6 mm diameter 30 cm, Target XL; Kalamazoo, MI: Stryker) was placed to fill the entire circumference between the lobes (Figure 1D).

Two following types of glass tubes were prepared: the stenotic model and the smooth model without stenosis. For each model, experiments were conducted under the following four conditions: (1) no embolization device, (2) only AVP II was placed, (3) a coil (6 mm diameter 30 cm, Target XL) was placed in the central lobe of AVP II (CIP technique), and (4) a coil (6 mm diameter 30 cm, Target XL) was placed between the proximal and central lobes of AVP II (macaron technique). After fixing the glass tubes horizontally, the injector was connected, and 100 mL of saline was injected at 20 mL per second. The amount of saline leakage from the side where the embolization device was deployed was measured. Each of these measurements was performed six times. From the eight groups, five groups were selected as follows: those without stenotic lesions, groups where the AVP II and macaron technique were deployed; from those with stenotic lesions, groups where the AVP II, coil in plug, and macaron technique were used. Comparisons between the two groups were conducted using the Mann-Whitney U test for all combinations. Analyses were conducted as two-tailed tests using JMP Pro version 17.2.0 (Cary, NC: SAS Institute), and the statistical significance level was set at p<0.05.

We applied this technique for the embolization of the IIA during endovascular aortic repair (EVAR) to prevent endoleaks. We retrospectively reviewed 11 patients who had undergone this embolization procedure with follow-up contrast-enhanced CT. Two board-certified radiologists independently evaluated the presence of recanalization and complications on postoperative contrast-enhanced CT and reached a final consensus. Informed consent and consent for publication were obtained from all the patients.

**Figure 2:**
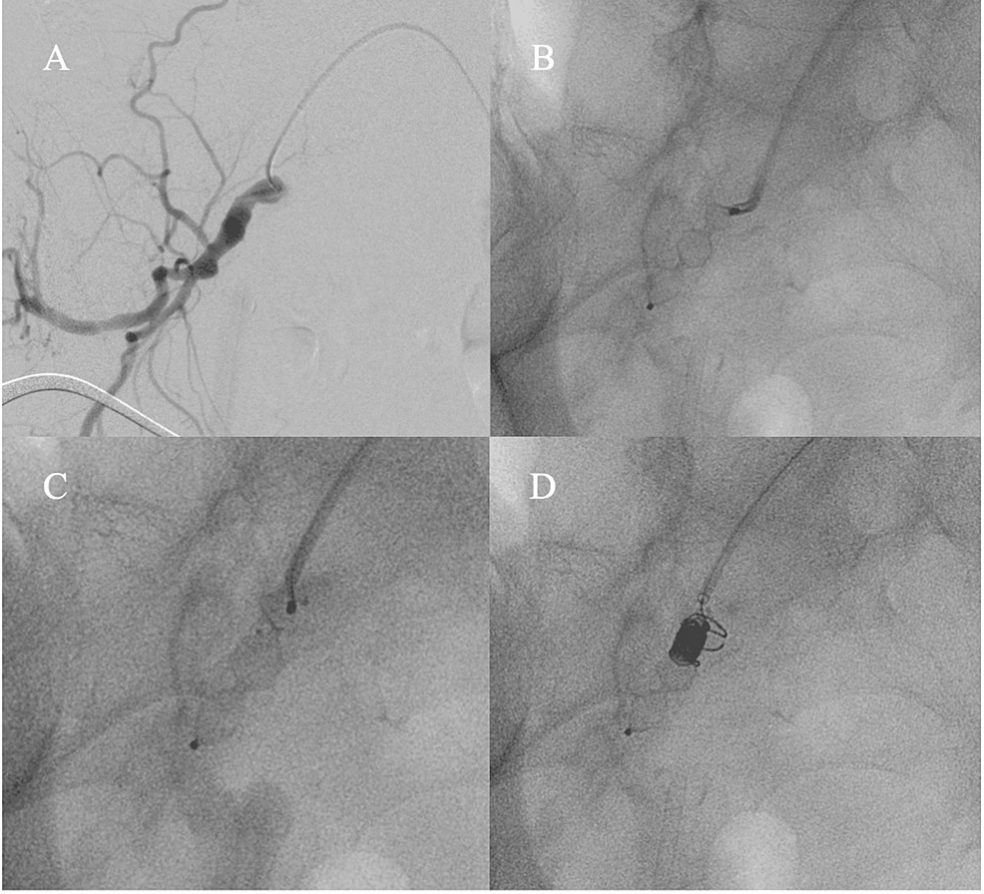
Sequential images of the embolization procedure. A 5 F guiding sheath was inserted via the left common femoral artery, and the tip of the sheath was placed on the right IIA (A). A 10-mm AVP II was deployed in the proximal portion of the right IIA (B). It was confirmed that the blood flow continued even after the AVP II was deployed. A 0.016-inch microwire was inserted through the side of the proximal lobe of the plug, and the microcatheter was placed in the space between the proximal and the central lobes of the plug (C). Prior to plug detachment, the space between the proximal and the central lobes of the plug was tightly filled with a detachable coil (Target XXL 8 mm 40 cm) (D). AVP: Amplatzer Vascular Plug

The following is the description of the representative case. For the embolization procedure, a 5 F guiding sheath (Destination; Tokyo, Japan: Terumo Corporation) was inserted via the left common femoral artery, and the tip of the sheath was placed on the right IIA (Figure 2A). A 10-mm AVP II was deployed in the proximal portion of the right IIA (Figure 2B). It was confirmed that the blood flow continued even after the AVP II was deployed. A 0.016-inch microwire (ASAHI Meister 16; Seto, Japan: Asahi Intecc Co Ltd) was inserted through the side of the proximal lobe of the plug, and the microcatheter (coiling support, Takarazuka, Japan: HI-LEX Medical) was placed in the space between the proximal and the central lobes of the plug (Figure 2C). Prior to plug detachment, the space between the proximal and the central lobes of the plug was tightly filled with a detachable coil (Target XXL 8 mm 40 cm, Kalamazoo, MI: Stryker) (Figure 2D). When the first coil was placed within the interlobe space, if the distribution of the coil was clearly biased or the coil density was deemed insufficient, a second coil was added. After confirming sufficient embolization, the AVP II was detached.

## Results

The amount of leaked saline in the experimental model is shown in Table 1. For all combinations of the five selected groups, pairwise comparisons were conducted, and the p-values were listed in Table 2. Since a total of 10 comparisons were made, the Bonferroni correction was applied, and p-values less than 0.005 were considered significant. When only the AVP II was deployed, the stenotic model had significantly more leakage than the smooth model (average 28 mL vs 19 mL, p=0.0044). The macaron technique significantly reduced leakage in patients with stenotic lesions compared with the AVP II alone (28 mL vs 10 mL, p=0.0045). The macaron technique had significantly less leakage than in CIP technique in the stenotic model (26 mL vs 10 mL, p=0.0044). The characteristics of the 11 patients are shown in Table 3.

**Table 1:** The amount of leaked saline in the experimental models. AVP: Amplatzer Vascular Plug

Experimental model	Deployed device	Amount of leaked saline (mL)						
		1st time	2nd time	3rd time	4th time	5th time	6th time	Average
Stenotic lesion (-)	No embolic device	48	50	50	47	47	50	49
	The AVP II	19	20	19	17	17	19	19
	Coil in plug	17	15	18	19	14	17	17
	Macaron technique	16	16	14	12	16	16	15
Stenotic lesion (+)	No embolic device	48	49	49	47	47	50	48
	The AVP II	27	28	28	28	26	30	28
	Coil in plug	26	27	26	27	27	21	26
	Macaron technique	9	11	9	12	9	8	10

**Table 2:** P-values for all pairwise comparisons among five groups with and without stenotic lesions using AVP II, coil in plug, and macaron techniques. AVP: Amplatzer Vascular Plug

Experimental model	Deployed device	Stenotic lesion (−)		Stenotic lesion (+)		
		The AVP II	Macaron technique	The AVP II	Coil in plug	Macaron technique
Stenotic lesion (−)	The AVP II	N/A	N/A	N/A	N/A	N/A
	Macaron technique	0.0040	N/A	N/A	N/A	N/A
Stenotic lesion (+)	The AVP II	0.0044	0.0041	N/A	N/A	N/A
	Coil in plug	0.0043	0.0040	0.0471	N/A	N/A
	Macaron technique	0.0044	0.0052	0.0045	0.0044	N/A

**Table 3:** The characteristics of the 11 patients. IIA: internal iliac artery; CIA: common iliac artery; AVP: Amplatzer Vascular Plug; M: male; F: female; Rt: right; Lt: left

Case number	Age	Sex	Location of the aneurysm	Site of the embolized IIA	Plug type and diameter (mm)	Coil type	Secondary coil diameter (mm)	Coil length (cm)	Follow-up time (days)
1	68	M	Lt CIA	Lt	AVP II (10)	DELTAFILL18	8	35	153
2	84	M	Aorta, Rt IIA	Rt	AVP II (10)	Target XXL	6	20	174
3	69	M	Rt CIA	Rt	AVP II (10)	Target XL, Target XL	8, 8	30, 30	240
4	86	M	Aorta, Rt IIA	Rt	AVP II (12)	Target XL, Target XL	8	30, 30	160
5	78	M	Aorta, Rt CIA, Lt CIA	Lt	AVP II (10)	Target XL	8	30	149
6	82	M	Aorta	Rt	AVP II (8)	Target XL	8	30	141
7	74	M	Rt CIA	Rt	AVP II (12)	Target XL, Target XXL	10, 12	40, 45	166
8	79	M	Aorta, Rt IIA	Rt	AVP II (10)	Target XL	10	40	215
9	78	M	Aorta, Rt CIA	Rt	AVP II (10)	Target XL	8	30	159
10	81	M	Aorta, Rt CIA	Rt	AVP II (10)	Target XXL	8	40	177
11	95	F	Aorta, Lt CIA	Lt	AVP II (10)	Target XL	8	30	145

The average number of coils used was 1.36. Contrast media was injected through the guiding sheath a few minutes after the coil was deployed, there was very little contrast flow into the peripheral side of the AVP in all cases, and in some cases, there was none at all. The addition of a coil to the interlobe dramatically reduced flow in cases where AVP II alone did not reduce flow at all after waiting several minutes. Follow-up enhanced CT (171 days after EVAR on average) showed good embolization in all patients without recanalization or leakage.

## Discussion

We developed and applied a coil packing technique between the AVP lobes for the embolization of IIAs with irregular lumens. Favorable embolization was performed in all 11 patients without recanalization.

Embolization using only an Amplatzer Vascular Plug (AVP) is associated with risks of residual leakage and recanalization [4,10-13]. Although these complications are rare, they can lead to serious consequences. The meshed structure of AVP has been considered one of the main causes [7]. We hypothesized another mechanism potentially leading to these issues; leakage may occur when AVPs are implanted in vessels with irregular lumen shapes due to insufficient adherence to the vessel. We conducted a preliminary experimental study to test this hypothesis and proved that there was significantly more leakage in the stenotic model than in the smooth model with AVP II. The most common solution for insufficient adherence of AVP is to add coils and/or glue before and after the plug. However, the disadvantage of this method is that embolization in short segments is difficult and if there is a vessel to be preserved proximal to the deployed site, it is difficult to add it without protruding. Our coiled packing technique between the AVP lobes overcomes these disadvantages and is expected to embolize vessels with irregular lumens more reliably.

The strengths of this technique are as follows: (1) similar to the CIP technique, microcoils can be added while maintaining high resistance to placement and migration, which are the advantages of AVP alone. In addition, the AVP anchoring technique reported by Onozawa et al. is also used, allowing stable microcatheter operation [14], and (2) similar to the double-balloon technique described by Yunaiyama et al., the local coil density can be increased with a small number of coils because the coil is filled in the space between the two lobes [15]. Further, the coil density can be increased by compressing the proximal lobe after the microcoil is filled. In IIA embolization, it has been reported that the CIP technique using the AVP I used an average of 3.8 coils, but our method used 1.36 coils [16]. Therefore, the cost-benefit of embolization with AVP, as compared to coil embolization, can be maintained [17], (3) because AVP II is shaped like beads and strings, by placing a coil in the gap, the coil can be filled up to the part closest to the center of the plug, and the blood flow through the mesh structure may be blocked, so that the same embolization effect as CIP may be expected. Even in cases with mild vascular lumen irregularity, there were cases in which the flow did not decrease easily even a few minutes after deployment with the AVP II. In such cases, the residual flow was dramatically reduced by adding one coil, at most two, with our method, and (4) our method is simple and does not require preloading techniques. It was necessary to pass the microwire beside the AVP and insert the microcatheter, but the insertion was possible without any difficulty in all cases. When passing a microwire, the proximal lobe of the plug was pulled and flattened by applying tension to the plug, making it easier for the microwire to pass through. When it is difficult to insert a microcatheter from the side of the AVP, the AVP can be retrieved, and we can switch to the CIP technique. Furthermore, this method can be used in combination with the CIP technique. In summary, the macaron technique provides more reliable embolization than AVP alone and may be highly effective in vessels with irregular lumen where AVP does not adhere well. It can be expected to reliably embolize vessels with poor AVP adherence that are difficult to embolize with the CIP technique. It should be noted; however, that this technique, unlike the CIP technique, is not applicable to AVP I. This method can be safely and reliably performed after AVP placement if necessary, when embolization is inadequate. We have used this technique in almost all cases in which AVP II is implanted and can recommend it as a standard approach. In particular, when the vessel to be implanted has an irregular lumen, when the coagulation function is abnormal, or when the vessel is not occluded after AVP implantation, this technique is well suited for use.

In our cases, the secondary coil diameter of the coil was chosen to be the same or slightly smaller than that of the AVP. With the macaron technique, the secondary coil diameter is not very important because the risk of coil migration is very low, but there is a risk that if you choose a secondary coil diameter that is too small, it will be unevenly packed in the interlobe, and the number of coils needed to fill all circumferences will increase. Also, if it is too large, there is a risk that it cannot fill in the interlobe, so it may be a good idea to use the same diameter as that of the AVP. Regarding the coil length, 30 cm (range: 20-45 cm) was used most frequently in our cases, and in all cases, the coil was able to fill in the interlobe. Rather, there were cases in which the filling appeared insufficient and additional coils were needed. Therefore, if the plan is to finish embolization with a single coil when using AVP II with a diameter of 8 mm or greater, it would be better to use a coil with a length of 30 cm or greater. In our cases, we employed bare platinum coils while applying the macaron technique. It is conceivable that fibered and hydrogel coils, if capable of flexible and dense packing, could also be effectively utilized. Such an approach may potentially result in more reliable embolization outcomes.

The drawback of this method is that it tends to have a longer embolization length than the CIP technique using the AVP I. The CIP technique using the AVP I may be better for short-segment embolization where the AVP II is difficult to place. Additionally, we used saline for the experimental vascular models. This would have introduced an underestimation of the effects of blood clotting. The reported CIP method uses multiple microcoils [7]. In the experimental model, embolization of the plug by the coils may have been insufficient. However, since the same coil was used for both the CIP method and the macaron technique, by comparison under the same conditions, the usefulness and cost-effectiveness of the macaron technique in the stenotic model were proven. The CIP method in the stenotic model showed an increase in leakage compared to the smooth model, suggesting that inadequate adhesion of the AVP was a factor in the increment in leakage.

## Conclusions

The AVP allows for embolization at the appropriate site with a low likelihood of migration; however, placement in irregular lumens may result in poor adhesion to the vascular wall, potentially leading to uncertain embolization. The use of the coil packing technique between the AVP lobes and/or the CIP technique may provide reliable embolization of various vessel forms.
